# AI-Augmented Teach-Back in Dentistry: From Patient Education to Verified Clinical Understanding

**DOI:** 10.7759/cureus.113774

**Published:** 2026-08-01

**Authors:** Abhi Thakkar, Bharani Kumar Bhattu, Chintan Desai

**Affiliations:** 1 Department of Dentistry, Camarena Health, Madera, USA; 2 Department of Dentistry, La Clinica De Familia, Las Cruces, USA; 3 Dental Public Health Resident, Tufts University School of Dental Medicine, Boston, USA

**Keywords:** artificial intellinge in dentistry, nlp chatboats, patient education, pediatric dentistry, teach-back

## Abstract

Oral diseases remain among the most prevalent chronic conditions worldwide, yet their prevention and long-term management depend heavily on patient understanding of self-care, maintenance protocols, and procedural risks. Existing educational programs have not solved this problem because most individuals do not understand dental health, and communication with patients remains poor, which results in poor treatment outcomes. The teach-back method functions as an evidence-based communication tool that helps staff members confirm that patients have grasped essential information, but dental practitioners use it inconsistently because it requires manual work and produces no measurable biological results. This review presents artificial intelligence (AI)-augmented teach-back as a communication system that unites dental evidence with healthcare field evidence to create a scalable system that maintains equity and enables verification. The review uses conceptual synthesis based on implementation science together with digital health and clinical communication research to study teach-back applications in pediatric, preventive, periodontal, surgical, geriatric, tele-dental, and public-health dentistry, which face three main challenges: standardization, workflow burden, and outcome measurement. Advances in natural language processing, speech recognition, semantic understanding scoring, explainable AI (XAI), and risk-adaptive communication systems have enabled teach-back to evolve from its original form as a recall-based educational method into a clinical process that can be audited and produces long-term results. This review uses three established frameworks together with the SALIENT AI-specific governance model to evaluate organisation readiness for implementation, their ethical safeguards, and their methods for achieving equity. The system provides three main advantages, including its ability to combine information from various fields, its design to match actual operational procedures, and its direct connection between communication verification methods and biological measurement results. This study depends on indirect evidence that comes from non-dental environments but does not include future dental research studies. The review presents communication as a clinical risk that can be modified and demonstrates how AI-augmented teach-back functions as a ready-to-use system for dental care improvement through better patient adherence and enhanced safety and equity distribution.

## Introduction and background

The worldwide prevalence of oral diseases is a major public health issue. These conditions affect billions of people despite medical and scientific progress. Dental caries in permanent teeth stands as a leading global health issue affecting more than 2.4 billion people throughout the world. Severe periodontitis affects almost one billion people worldwide, and tooth loss creates ongoing life-quality challenges for different populations [1-3]. These conditions are chronic and recurrent, often requiring lifelong prevention and maintenance rather than a definitive cure. Oral diseases represent an unfair distribution of health problems because they mainly affect children, senior citizens, and people from developing nations who face obstacles in obtaining dental care [4]. These diseases continue to affect many people despite the existence of effective medical treatments, which shows that medical care availability does not guarantee proper treatment results and thus requires additional healthcare system reforms and disease prevention methods [5].

Within this context, communication failure in dentistry must be reframed as a clinical risk factor rather than a mere educational gap. Research shows that patients who fail to understand medical information will not follow treatment plans, which leads to worsening of their health conditions. Analysis of extensive population data demonstrates that individuals with poor health literacy skills develop more dental caries, periodontal disease, and tooth loss because they fail to maintain proper oral hygiene and they avoid dental care visits [6]. Research on implants, spanning multiple years, demonstrates that patients who do not follow maintenance guidelines will develop more cases of peri-implant mucositis and peri-implantitis [7]. Systematic reviews show that patients need to follow their supportive periodontal therapy treatments to maintain their health because skipping treatment leads to insufficient management of the condition [8]. Evaluation of implant failures shows that patients who do not follow their post-treatment care instructions will experience more surgical problems that lead to implant failure [9]. The complete set of findings indicates that miscommunication is a modifiable clinical risk factor that functions similarly to insufficient treatment planning and inadequate patient monitoring.

Oral health literacy refers to the ability of individuals to obtain, process, and understand basic oral health information necessary to make appropriate health decisions. Research demonstrates that dental patients across demographics face difficulty understanding written information, particularly affecting those who lack access to dental care: Most rural outreach patients experience poor oral health literacy, which leads them to misunderstand dental health information and results in inadequate dental care and preventive actions [10]. Research shows that people with low oral health literacy develop stronger dental anxiety, which makes them avoid dental care, thus creating poor treatment adherence [11]. Analyses reviewing multiple studies indicate that traditional educational methods that focus on direct instruction help patients learn information quickly but do not produce permanent behavioral changes in elderly people or those who struggle with reading [12]. Research also shows that standard educational techniques fail to achieve desired oral health results because patients need tailored communication methods that match their literacy levels to succeed.

Teach-back operates as a formal communication method that enables patients to demonstrate their knowledge by rephrasing information, which helps prevent misinterpretations. Medical research has shown that this method shows strong clinical results. Systematic reviews show that teach-back methods help patients understand their health better and maintain their self-care activities and medication use in chronic disease treatment while decreasing the occurrence of safety-related adverse events [13]. Research on elderly surgical patients shows that teach-back methods produce better patient understanding and lower anxiety levels than traditional educational methods [14]. The medical evidence supporting this practice stands strong, yet dentists have not implemented it consistently. Limited research studies that test teach-back in pediatric dentistry show that this method helps children remember dental care instructions right after learning them, but it does not provide enough evidence because it involves few participants and tracks their behavior for only a short period [15]. Although teach-back is used in medical settings to confirm patient understanding, its routine application in dental clinics remains limited.

Teach-back functions as an effective communication method, but dentistry faces challenges when it comes to applying this method on a large scale. Research shows that definitions and implementation approaches vary for this topic; awareness of the method has increased but faces ongoing challenges with time demands at the dental chair, differences among providers, and unstandardized treatment protocols [16]. The medical field promotes teach-back as a safety measure to improve patient understanding, but staff resists this approach because it creates workflow interruptions and does not include automated systems to confirm patient comprehension [17]. Research shows that teach-back functions properly, but dental clinics need automated systems and standardized evaluation methods to support routine implementation at scale.

Artificial intelligence (AI) offers potential too to address patient communication scalability problems by working alongside clinicians instead of taking their place. ChatGPT-based systems operate through natural language processing chatbots that enhance oncology patient understanding while minimizing communication problems [18]. Jabeen and Saji indicate that AI-generated patient education materials provide better understanding than standard educational materials through user-friendly content [19]. Giguere et al. demonstrate that AI-based decision support systems enable patients to obtain better comprehension through interactive educational tools that help them make better choices [20]. Studies have established that chatbots together with virtual assistants enable hospitals to perform triage activities and educate staff members to boost their operational performance, but their implementation requires both public trust and regulatory authorization [21]. The Virtual Healthcare Bot represents a person-centered system that uses AI to decrease healthcare provider workload while maintaining personalized patient care [22]. To date, however, dentistry has largely focused AI applications on diagnostics, leaving patient communication comparatively underexplored.

Current evidence indicates a major gap in the existing body of research. Medical professionals use teach-back as their established communication method, but dental care providers need to evaluate how AI systems might solve their current communication problems. Unclear communication between dental teams and patients can lead to poor treatment outcomes because patients do not follow medical advice correctly. The available solutions used by many organizations fail because they do not maintain sufficient levels of standardization. This review investigates dental teach-back evidence and assesses AI-based teach-back systems that generate standardized communication, scale across patient populations, and support equitable communication access. It combines dental education evidence with health literacy research and medical AI communication systems to create a complete theoretical and practical framework for AI-augmented teach-back application in dental practice.

## Review

Teach-back in dentistry: evidence, applications, and unresolved gaps

Dental professionals now use teach-back as their main communication method, which helps patients overcome health literacy difficulties and helps reduce preventable clinical errors from occurring. The teach-back method transforms information delivery into an interactive process that requires patients and learners to explain the material in their own words, thereby demonstrating their understanding. The dental field uses this method to teach children, help patients maintain periodontal health and implants, and educate dental professionals. Research shows that people achieve greater understanding, improve memory retention, and follow instructions better during the first period of their treatment. However, research results are fragmented because they include small numbers of participants and depend mostly on personal evaluations and actions instead of objective measurements. The dental field lacks a standardized method to evaluate comprehension because clinicians do not use objective tools; this prevents them from using teach-back effectively in their work to generate consistent outcomes and achieve permanent clinical benefits.

Pediatric and school-based dentistry

Children, together with their caregivers, face multiple obstacles that affect their ability to read, which can limit teach-back’s effectiveness for use in pediatric dental care. Research in schools and through outreach programs shows that students achieve better comprehension during the initial period when using teach-back and other similar interactive teaching methods. One study conducted a controlled educational intervention with 12- and 13-year-old students, which showed that teach-back produced better results for brushing step recall than the Tell-Tell-Tell and Ask-Tell-Ask methods [15]. Active recall methods proved effective in enhancing preventive instructions that students receive after receiving their initial training, according to the research results.

Research from community outreach programs using observational study methods validates these findings. Immediate comprehension of preventive instructions becomes better through basic recall methods, which benefit people who have limited knowledge about dental health [10]. People who cannot read well tend to develop dental anxiety, which leads them to avoid dental care, according to Badran et al., who highlight the need for dental teams to develop alternative communication methods for patients with limited literacy [11]. Teach-back methods minimize the communication obstacles that create the most problems for children who need extra support.

Despite these early gains, the durability and scalability of pediatric teach-back interventions remain limited. Systematic reviews of oral-health education programs demonstrate that conventional interventions yield short-term improvements in knowledge, such as fluoride use and diet counseling, but fail to sustain behavioral change over time [12]. Interactive and game-based teaching methods produce identical results because students show immediate improvement in comprehension; their dental plaque score decreases temporarily before returning to baseline after the program ends [23]. The teach-back method across healthcare settings improves patient understanding, but research has faced obstacles due to limited numbers of participants and prolonged training periods [16]. The pediatric teach-back program conducts occasional sessions, which require funding, but it is not a normal part of school activities, and it lacks ongoing support to maintain its effects.

Periodontal and implant care: teach-back in adherence-dependent treatments

Periodontal and implant results depend on patient compliance, so teach-back functions as an effective method to reduce risks during patient education sessions. The first stages of treatment show modest improvement according to evidence from quasi-experimental and observational research when medical teams use interactive communication methods with simplified information delivery. The teach-back educational method has demonstrated its effectiveness for implant surgery patients to follow postoperative instructions immediately while building patient confidence and reducing early medical issues [24]. Research demonstrates that teach-back improves patient involvement during essential perioperative periods that last for a limited time.

The same patterns appear in the treatment of periodontal disease. Research on periodontal home-care therapy shows that patients who maintain brushing and interdental cleaning activities experience initial reductions in plaque and gingival measurements, but their adherence tends to fade when they lack ongoing support [25]. Research on audio-visual aids for periodontal education shows that students learn better and maintain plaque control for short periods, but the benefits vanish after three months [26]. Interactive learning methods help students achieve better initial adherence, but these methods fail to produce permanent adherence outcomes.

Teach-back in dental education: training benefits and assessment limitations

Teach-back has become a teaching method in dental education, and it has been incorporated in their communication and professionalism courses rather than as an independent subject. Studies about curriculum development demonstrate that active learning combined with teach-back principles improves both student results and their ability to engage patients [27]. Simulated patient studies through prospective cohort surveys demonstrate that specific communication training programs boost student performance while also enhancing instructor assessments of student competence to support teach-back effectiveness in strengthening student-patient connections [28]. Video-based and feedback-oriented approaches similarly demonstrate that interactive communication methods promote self-reflection and perceived learning gains among dental students [29].

Chairside teaching models employ blended learning approaches that include problem- and case-based learning and structured seminars to prove real-world usefulness. A randomized controlled trial shows students who received interactive chairside instruction develop better confidence and communication abilities [30]. Student-perception studies show that clinical teaching requires instructors to lead effectively, yet different teaching methods are used by different faculty members [31].

Assessment serves as a structural weakness in educational settings because it provides pedagogical advantages to students. Objective Structured Clinical Examination (OSCE) rubric psychometric analysis shows large differences between rater responses and rubric categories, which contradicts the belief that communication skill assessment remains objective [32]. The evaluation of observed assessments throughout time has revealed that evaluators continue to demonstrate bias, while their assessment methods remain inconsistent [33]. System-level dental-care quality measurement evaluations by systematic reviews show that dental organizations lack standardized assessment tools to evaluate communication skills, which leads to the development of individual assessment systems [34].

Structural limitations of manual teach-back in dentistry

Research shows that dental teach-back provides effective short-term results, yet the current educational framework prevents its extensive application, which also limits its development into a sustainable teaching method. Studies mainly focus on short-term results, which include patient recall during the same visit and initial treatment compliance, but they fail to monitor patients beyond several weeks to a few months, which prevents assessment of long-term knowledge retention and behavior maintenance [13,24]. Teach-back protocols exist in multiple forms that lack standardization because they use different delivery methods, levels of intensity, and staff members, thus making comparisons and replication difficult [13]. The system lacks digital or automated delivery systems, which makes its implementation process slow because providers must perform every step manually [35]. Outcome evaluation depends on unvalidated comprehension assessments because teach-back uses self-reported confidence, clinician judgment, and adherence tracking as its main evaluation tools [13,24]. Finally, adult and geriatric dental populations remain underrepresented despite bearing the highest burden of chronic disease and consent complexity [35].

Patient understanding lacks standardized evaluation methods, which affects all pediatric programs together with periodontal and implant maintenance and dental education programs. The reviewed studies fail to use any validated cognitive-load metrics, error-pattern analysis methods, or structured comprehension scoring systems. Research teams have used binary confirmation together with patient self-assurance, clinician evaluation, and monitoring of patient behavior to determine their understanding. The use of proxy indicators hides unexpressed confusion, which prevents scientists from comparing results or applying proven communication methods to larger populations.

Teach-back produces immediate improvements in dental knowledge retention and patient participation, but its advantages do not result in long-term patient compliance or population-wide health improvements. The system faces structural limitations, which include manual delivery requirements, provider variability, chairside time constraints, and the absence of objective verification systems and reinforcement tools. Standardized measurement systems need to be created together with scalable detection and correction systems, which will make teach-back effective for long-term use across broad healthcare environments. Research must identify the current measurement gap because this gap prevents teach-back from developing into a long-lasting communication system that benefits dental patients through equal care and enhanced organizational efficiency. These limitations are consistent across pediatric, preventive, periodontal, surgical, geriatric, tele-dental, and public-health settings, as summarized in Table 1.

**Table 1:** Evidence landscape of teach-back in dentistry: domains, outcomes, and measurement gaps RCT: randomized controlled trial; BOP: bleeding on probing

Dental Domain	Communication Modality	Study Type	Primary Communication Outcome	Verification of Understanding	Biological/Clinical Outcomes	Follow-up	Key Evidence Gaps	Value Added by AI-augmented Teach-Back	References
Pediatric/School-based dentistry	Conventional education; manual teach-back	Educational RCTs; observational	Immediate recall; short-term comprehension	Manual teach-back; self-report	Short-term plaque reduction; hygiene behavior	Immediate–three months	Effects transient; no objective comprehension scoring; no long-term outcomes	Standardized comprehension scoring and automated reinforcement beyond single sessions	[12,15]
Preventive dentistry (adults)	Counseling; manual teach-back; digital education	Cross-sectional; systematic reviews	Knowledge, confidence, perceived understanding	Self-report; clinician impression	Rare; adherence proxies only	Immediate–weeks	Education weakly linked to biology; literacy mismatch unmeasured	Literacy-adaptive explanations with semantic verification before behavior failure	[11,13]
Periodontics (supportive care)	Conventional education; manual teach-back	Retrospective; prospective cohorts	Compliance; maintenance understanding	Behavioral proxy only	Plaque Index; Gingival Index; BOP; tooth loss	Six to 11 years	Comprehension assumed, not measured; education non-standardized	Direct linkage of verified understanding to long-term biological stability	[36]
Implant dentistry	Postoperative counseling; manual teach-back	Retrospective; observational	Instruction recall; confidence	Self-report	Implant failure; peri-implant disease	One to 11 years	Patient factors identified but comprehension never quantified	Early detection of misunderstanding in high-risk maintenance protocols	[37,38]
Oral surgery/Informed consent	Standard consent; teach-back; digital consent	Prospective studies; reviews	Risk recall; consent quality	Manual teach-back	None (ethical/legal focus)	24 hours–seven days	Rapid recall decay; no audit-ready comprehension metrics	Risk-adaptive explanations and objective, documentable consent verification	[17,39]
Geriatric dentistry	Conventional education; caregiver counseling	Ethical analyses; observational	Understanding; anxiety; decision capacity	Clinician impression	Periodontal progression; hygiene adherence	Variable	Cognitive decline unaccounted; caregiver role inconsistent	Simplified, multimodal teach-back with longitudinal comprehension tracking	[40,41]
Tele-dentistry	Verbal instruction; teach-back	Reviews; educational trials	Recall; adherence	Verbal teach-back only	Rarely assessed	Immediate–short term	Loss of non-verbal cues; no objective verification	Restores feedback loops via speech-to-text and semantic scoring	[42,43]
Public health dentistry	Mass education; community teach-back	Mixed-methods; Program evaluations	Recall; confidence	Manual teach-back	Population-level adherence proxies	Short-term	Literacy mismatch; manual delivery not scalable	Multilingual, scalable comprehension verification at population level	[44,45]
Cross-domain synthesis	Manual teach-back (all domains)	Systematic reviews	Short-term comprehension gains	Mostly none	Rare and inconsistent	Short-term	No standardized comprehension metrics; weak education→biology linkage	Unified verification, scalability, and longitudinal reinforcement across domains	[13,46]

AI in health communication: from information delivery to understanding verification

AI applications in patient communication now extend that go beyond basic information delivery to enhance patient understanding, treatment compliance, and service expansion for chronic illness management. Medical fields use AI-driven systems that combine natural language processing with conversational interfaces and explainability frameworks to overcome the basic operational problems that disrupt manual teach-back communication methods. Evidence demonstrates that dental AI communication systems with human interaction capabilities will work successfully in clinical dental practice.

Natural language processing chatbots and conversational agents in chronic disease education

Natural language processing-enabled chatbots function as successful educational tools that help patients with chronic diseases by enhancing their understanding and compliance with treatment while expanding providers’ reach to multiple patient groups. Systematic reviews and experimental studies demonstrate that chatbots transform difficult medical information into simple content through interactive conversations that enable users to actively participate in educational material. The research on natural language processing chatbots by Aggarwal et al. demonstrates that chatbot-based educational programs promote improved understanding in patients while enhancing their medication adherence by 15%-20% beyond what standard educational methods achieve [47]. Casu et al. have shown that mental-health chatbots enable users to grasp therapeutic information more effectively, while users completed almost 70% of the program, which demonstrates the chatbots’ ability to sustain user engagement throughout lengthy timeframes [48]. Research teams have confirmed these results through their work with specialized applications that target particular professional fields. Research in oncology demonstrates that patients who receive chatbot-based education show a 25% increase in their ability to understand information and read better, which has led to improved adherence to their treatment plans [49]. The research findings from ophthalmology applications mirror these results; Ittarat et al. have found that patients better understand medical terms, and their medication compliance has improved by 10%-15% for glaucoma and diabetic eye disease treatment [50]. Kurniawan et al. have demonstrated that in chronic disease management, patients increase their knowledge by 20%-30%, while they achieve a 12% improvement in diabetes self-management compliance [51]. Reis et al.’s findings show improved adherence between 18% and 22%, whereas Hou et al. have demonstrated that patients could keep their hypertension knowledge at about 20% [52]. The studies demonstrate that natural language processing chatbots function as cost-effective tools to help patients with chronic diseases better understand their conditions while following medical guidance.

AI-supported consent and shared decision-making 

AI-powered communication systems may be particularly useful for teach-back applications that include teaching patients and working together to reach common decisions. Chau et al. have identified algorithmic opacity as the main factor that causes patients to struggle with understanding medical procedures because they need to receive ongoing educational support instead of a single consent form [53]. The authors support three fundamental design elements based on regulatory guidance and human-AI interaction research: layered explanations, adaptive disclosure, and repeated clarification loops. High-risk clinical environments have produced data that prove that these documented issues remain valid. Froicu et al. have revealed that oncology patients continue to experience consent problems because they do not understand how AI systems function in cancer diagnosis and treatment selection, which results in patient confusion and decreased medical trust [54]. Tarantini et al. have found that standard consent practices fail to meet the needs of complicated heart procedures, yet AI-powered counseling systems that work with transparency help patients understand their individual risks better while decreasing their uncertainty about treatment decisions [55]. Park et al. have demonstrated that AI systems, which users can understand in detail, help them make better decisions while gaining system acceptance; however, users of non-transparent AI systems develop stronger resistance and anxiety [56].

Conversational systems provide additional advantages that build upon existing benefits. The prostate cancer education AI chatbots developed by researchers have enhanced patient understanding by 20%-30% while lowering patient decisional conflict and delivering user-friendly interfaces [57]. Urology studies have shown similar results in patient engagement and shared decision-making through the combination of AI chatbots with medical staff counseling [58]. The Consent 2.0 model and other conceptual frameworks demonstrate that current consent forms lack compatibility with AI-driven medical care systems because they need to be updated continuously to protect patient autonomy during an evolving consent process [59].

Speech-based AI and comprehension detection

AI systems that operate through speech-based interfaces address the main challenge that stops patients from demonstrating their understanding of educational material. Voice-enabled AI systems process patient speech immediately to identify when patients hesitate, make errors, or fail to finish their statements, which activates an automatic system for clarification. The oncology communication systems that Loeffler and his team developed in 2025 improved recall accuracy and decreased decisional conflict because they identified patient confusion during medical discussions [60]. Zellou and Holliday have shown that NLP-based spoken dialogue systems correctly identify conversation breakdowns, which they then use to generate corrective feedback [61].

Research on technical development shows that the project will succeed in its implementation. The Bidirectional Encoder Representations from Transformers (BERT) based voice interface system uses semantic scoring to evaluate how well patients restate information, which enables automatic assessment of comprehension through dynamic feedback mechanisms [62]. Deep-learning models operating on large-scale multilingual data sets show the ability to identify misunderstanding predictors, which results in improved comprehension for various population groups [63]. The research conducted in educational settings proves similar results because Google Assistant, along with other intelligent personal assistants, fixed user errors instantaneously, which led to better response accuracy [64]. The research shows that voice assistants help teach-back methods evolve from simple manual repetition to automated systems that monitor student comprehension at scale.

Explainable AI (XAI) and health literacy

XAI stands as a basic requirement that enables people to trust health communication systems while understanding and using them effectively in environments where literacy support is necessary. User literacy levels determine the amount of explanation that should be provided in human-centered systems to prevent users from becoming overwhelmed by complex information. The PINXEL framework introduced by Panagoulias et al. creates explanations that change according to patient reading ability to enhance both understanding and trust perception [65]. They have proven that machine learning studies, which combine the Technology Acceptance Model with the Rapid Estimate of Adult Literacy (REALM) literacy assessment, demonstrate that explanations matching literacy levels help users understand content better and accept it more; however, unclear outputs reduce their interest in the content [66].

Evidence that focuses on clinicians leads to identical findings. The systematic review conducted by Rosenbacke et al. demonstrates that AI systems with clear explanations and understandable designs lead to better clinician trust and system adoption, but models with insufficient explanations prevent their application [67]. Research that analyzes studies has identified explainability as the fundamental requirement for healthcare organizations to operate ethical AI systems [68]. Research conducted at the patient level shows that medical AI trust develops through doctor-patient interactions, and patients become more trusting when doctors provide explanations that match their comprehension abilities [69]. Findings demonstrate that teach-back models using AI systems need explainability as their fundamental requirement to achieve verified understanding and maintain continuous user involvement.

Conceptual framework: AI-augmented teach-back as a verifiable communication system

Teach-back with AI augmentation functions as a complete system that turns patient education into an active process, providing customized health information and confirmation of patient understanding. The framework consists of five interrelated elements: (i) explanation generation that adapts to literacy levels, (ii) precise documentation of how patients restate information, (iii) automated evaluation of semantic understanding, (iv) self-executing clarification systems, and (v) explanation depth based on risk assessment. The system resolves three fundamental weaknesses of traditional teach-back, which include staff-dependent variations, the time staff must invest, and an inability to track patient development. However, it does allow medical staff to keep their supervisory position. 

AI-generated, literacy-adaptive explanations

AI-generated explanations function as the fundamental system that enables teach-back to scale because they create educational materials that match patient health knowledge levels instead of using basic content simplification. The XAI system modifies its output by changing its vocabulary, sentence construction, and information amount according to the detected or assessed literacy abilities of users. The research team led by Panagoulias has shown that the technology acceptance model combined with REALM, in medicine, achieves better results when machine learning determines explanation depth, leading to improved comprehension and user acceptance compared to fixed educational content [66]. The PINXEL framework implements literacy-based explainability through its system, which controls explanation delivery methods including text, voice, and visual signals, while doctors monitor the process to enhance trust development in medical training simulations [65]. Medical education becomes more effective through health-care NLP systems that use adaptable dialogue systems to customize their explanations based on patient needs, while clinicians stay involved in the communication process [70]. Large language models (LLMs) undergo multiple assessment stages that demonstrate that progressive disclosure methods achieve better results than unchanging content because they improve patient and clinician memory retention, understanding, and trust development [71].

Research comparing patient information sources demonstrates that AI-generated explanations produce more readable content that users understand more effectively than standard leaflets while simultaneously reducing their need for decision-making [72]. Results demonstrate that teach-back requires literacy-adaptive AI explanations to function as a fixed educational system that forms the basis for their operational success. 

Patient restatement capture via speech recognition

AI-augmented teach-back requires correct recording of patient restatements because it depends on transcription of patient explanations instead of using clinician dictation for comprehension assessment. Modern automatic speech recognition (ASR) systems now demonstrate sufficient clinical performance based on current evidence. Research from Ng et al. indicates that advanced ASR systems generate word error rates between 10% and 15% during their analysis of organized clinical dialogues, which support semantic processing but fail to produce exact transcriptions [73]. ASR systems perform well when used in real-world settings that involve spontaneous speech, various speech forms, and common background noises that patients use for their teach-back information repetition [74].

Recent studies demonstrate that unprocessed transcription accuracy fails to serve as the correct standard for assessing patient interactions with health-care staff. Minor errors that do not change the fundamental meaning of the text allow ASR systems to perform clinical comprehension assessments instead of legal document processing [75]. The development of multilingual ASR systems that use language models has improved system reliability for all users while solving dental practice problems regarding patient-care equality and health information understanding [76]. Research data prove that speech-to-text technology operates successfully to record patient responses that AI-enhanced teach-back systems identify as restatements.

Semantic understanding scoring and error detection

A central limitation of conventional teach-back is the absence of objective methods to determine whether a patient’s restatement reflects true understanding rather than superficial recall. The established solution for semantic understanding evaluation has become possible through NLP developments and LLM progress that enable meaning alignment scoring, concept coverage assessment, and critical information omission detection. A study conducted in medical education showed that LLM-based semantic grading methods achieved human-level agreement with expert evaluators when they graded 2,288 responses. The LLM approach showed better performance than keyword-based methods because it could identify student comprehension errors and incomplete knowledge [77]. Evidence has shown high levels of agreement between LLM-generated semantic scores and expert grading across medical fields (r = 0.85). A system has demonstrated enhanced ability to detect absent safety-essential concepts during medical document analysis [78].

A systematic review has demonstrated that semantic similarity and concept-coverage models show better agreement with clinician assessments than surface linguistic metrics do. The authors have identified these systems as decision-support tools instead of independent evaluation systems [71]. The patient-facing validation process has confirmed that AI-generated educational materials provide better semantic coverage of vital safety information than leaflets created by clinicians, according to human-verified comprehension standards [72]. The results prove that natural language explanations can be used to measure comprehension through an objective method that enables standardized and auditable teach-back assessment.

Automated clarification and feedback loops

Automated clarification loops turn teach-back into an ongoing learning system that goes beyond single interactions to achieve operational status. The field of intelligent tutoring and health-care education demonstrates that adaptive feedback systems that detect student confusion lead to improved learning outcomes and better information retention. As’ad has demonstrated that tutoring systems based on generative AI technology can detect mistakes in student answers, while students also enhance their explanation skills and assess their understanding, which leads to superior learning retention compared to conventional educational methods [79]. Research using longitudinal randomized methods shows that students who receive daily adaptive feedback will develop better self-regulated learning abilities while maintaining their understanding of the material [80].

Studies have demonstrated that speech-based adaptive feedback systems operate effectively in health-care settings with noisy environments, while their iterative clarification process leads to better memory retention [81]. LLMs that operate under human-in-the-loop supervision systems can detect instances of partial understanding and perform targeted reformulations that track the system’s advancement through transparent documentation that complies with regulatory standards [82]. Research demonstrates that automated clarification loops function as a scalable solution that enhances teach-back methods, while clinicians maintain their decision-making authority.

Risk-adaptive explanation depth

Risk-adaptive explanation depth follows standard clinical communication protocols because medical staff must give different levels of information depending on the complexity of procedures and the expected severity of harm. Surgical consent literature demonstrates that patients achieve better understanding and higher satisfaction when medical professionals provide information that matches the level of risk involved. Rimmer has proven that using stratified consent with basic explanations for low-risk procedures and detailed discussions for high-risk interventions could result in better patient understanding while maintaining the correct level of explanations [83]. Research shows patients want stratified information that they can access immediately because they will pay more attention when they understand their medical procedures involve high risk [84]. Systematic evidence further supports this approach, with reviews concluding that risk-aligned, layered explanations outperform uniform communication strategies in complex clinical scenarios [68]

Medical practitioners who perform surgical operations modify their explanation approaches based on risk calculator results and patient health status, but patients show better satisfaction when doctors communicate according to their estimated risk [85]. These findings establish a solid research basis that supports the application of risk-based explanation depth in AI-augmented teach-back frameworks. The functional architecture of AI-augmented teach-back, including explanation generation, semantic verification, and auditability, is summarized in Table 2.

**Table 2:** Functional architecture of AI-augmented teach-back: components, outputs, and governance NLP: natural language processing; LLMs: large language models; XAI: explainable AI; ASR: automatic speech recognition

Teach-Back Function	AI Technique	Primary Inputs	Primary Outputs	Clinical & Implementation Value	Ethical/Governance Safeguards	References
Literacy-adaptive explanation delivery	NLP-based LLMs; XAI	Patient language, literacy indicators, clinical risk	Tailored explanations (text/voice/visual) matched to literacy and risk	Improves comprehension without increasing chairside time; supports equity	Human-in-the-loop review; adjustable explanation depth	[65,66,72]
Patient restatement capture	ASR; multilingual ASR	Patient verbal restatements	Accurate transcription for analysis	Enables scalable teach-back in clinic and tele-dentistry	Accent bias monitoring; multilingual validation; clinician override	[73,74,86]
Comprehension verification	Semantic similarity scoring; concept-coverage models (LLMs)	Transcribed restatements; predefined safety concepts	Quantitative comprehension score; missing-concept flags	Replaces subjective judgment with standardized, auditable verification	Decision-support only; explainable scoring logic	[71,77,78]
Clarification & reinforcement	Generative AI with adaptive feedback rules	Error patterns; partial understanding signals	Targeted re-explanations; iterative feedback	Detects silent misunderstanding; supports longitudinal retention	Clinician supervision; controlled iteration thresholds	[79,80,82]
Risk-adaptive explanation depth	Risk-stratification and layered disclosure algorithms	Procedure complexity; patient risk profile	Stratified explanation intensity	Improves consent quality; avoids under- or over-disclosure	Patient control; auditability; consent alignment	[68,83,84]
Documentation & audit trail	Secure NLP summarization; compliance logging	Interaction logs; comprehension scores; timestamps	Audit-ready records of verified understanding	Reduces medico-legal risk; supports quality assurance	Data minimization; explicit consent; privacy-preserving processing	[87,88,89]

Clinical applications of AI-augmented teach-back across dental domains

Multiple dental fields benefit from AI-augmented teach-back because communication problems create common clinical risks that vary depending on treatment complexity, patient needs, and medical environment. The clinical goals of preventive dentistry, implant therapy, surgery, geriatrics, tele-dentistry, and public health vary, but these fields depend on patients to correctly understand and continue following their instructions. The upcoming sections show how AI-augmented teach-back resolves particular communication problems in different fields through its ability to confirm patient understanding, uniform teaching approaches, and a method of supporting medical professional choices instead of substituting their expertise.

Preventive dentistry: verifying instruction-dependent self-care

Preventive dentistry faces its most significant risk of communication breakdown because its success relies on patients to correctly grasp and regularly perform their daily self-care activities instead of receiving occasional clinical treatment. Evidence consistently shows that preventive strategies fail primarily due to misunderstanding rather than lack of access. Patients frequently misinterpret fluoride use, brushing and flossing techniques, optimal frequency and timing, and the clinical significance of caries risk in the absence of symptoms. Research by Badran et al. and Ramos-Gomez and Tiwari demonstrates that low oral health literacy leads people to develop dental anxiety because they avoid preventive care and fail to follow their dentist’s recommendations [11, 90].

Teach-back functions as an operational tool that helps health-care providers verify patient understanding by having patients restate their brushing, flossing, and fluoride-use instructions using their own words to identify hidden comprehension issues that would not be detected otherwise. Research shows that teach-back methods help patients remember information better, while they also understand it better and follow instructions more effectively in the short term across medical facilities. The practice of using teach-back as a standard procedure in dental clinics remains unpredictable because dental professionals have different availability and they spend different amounts of time with their patients [13]. The teach-back process is substantially enhanced through AI, which produces explanations that match patients' literacy levels while performing automated semantic evaluations of patient restatements and establishing automated clarification procedures to handle situations when patients fail to understand information. Research shows that AI-generated educational materials in dental clinics help patients reduce their anxiety levels, while they better understand preventive dental care and restorative dental procedures, which proves AI serves as an additional tool for medical professionals instead of taking their place [36].

Periodontics and implant dentistry: adherence-sensitive long-term care

Preventive care focuses on daily routines, but periodontics and implant dentistry pose greater risks when people fail to understand their importance because patients need to follow complex maintenance protocols for clinical success. The practice of implant therapy enables patients to become active partners in managing their treatment results through dedicated oral care, scheduled dental checkups, and proper management of their health and behavior risks. Clinical evidence shows that early implant failures occur because of patient-related factors that have no connection to surgical methods. Research analyzing large amounts of data has found that poor oral hygiene, together with smoking, systemic disease, and implant location, determines which patients may experience early implant failure [37]. The risk of failure becomes more severe when patients have osteopenia or osteoporosis because these conditions affect their ability to receive subsequent implants, which requires ongoing patient education about their condition and specific care plans [38].

Periodontitis treatment requires patient adherence to achieve permanent success during supportive periodontal therapy because patients who fail to follow instructions will experience higher tooth loss than those who receive professional care. The teach-back method functions best in this environment because it enables medical staff to confirm patient understanding of complex maintenance protocols and early peri-implant disease symptoms that patients generally misinterpret during standard treatment [36]. The teach-back system receives additional support from AI, which creates uniform post-implant education programs, measures patient comprehension of complex medical instructions, and detects dangerous misunderstandings at the initial phase. Evidence in the dental field indicates that AI-generated educational materials enhance semantic comprehension and reduce patient anxiety, which shows AI technology should function as a safety system for periodontal and implant care that requires patient adherence rather than replacing professional medical decision-making [91].

Oral surgery and informed consent: risk communication and recall decay

Oral and maxillofacial surgery requires precise communication because its informed consent process leads to ethical, legal, and safety challenges. Studies have revealed that patients may give consent without fully understanding the information, and their memory of the information fades quickly. A prospective study has shown that more than 60% of oral-surgery patients forgot about major surgical risks after 24 hours, and their knowledge about alternative treatments and post-surgery duties dropped even more by the seventh day, even though they believed they understood the instructions [39]. Emergency surgical environments experience a similar problem because consent is obtained from patients without understanding their situation [92].

Teach-back solves this ethical problem by making patients demonstrate their understanding of risks and responsibilities using their own words, which leads to better comprehension, memory retention, and improved decision-making when compared to traditional consent procedures [17]. The teach-back method continues to be applied sporadically, yet health-care facilities do not keep track of its execution through verifiable records. The consent process becomes more efficient through AI-augmented tools that provide explanations that match risk levels, provide information at suitable reading levels, and create audit-ready documents to show patient comprehension without taking over clinical decision-making [93]. The combination of digital consent systems with smartphone-based platforms enables patients to remember information better, while they experience lower anxiety and higher satisfaction levels, which proves AI systems can function as scalable support systems for clinicians who deliver informed consent during oral surgery [94].

Geriatric dentistry: cognitive vulnerability and caregiver-mediated understanding

Geriatric dentistry requires advanced procedures because patients face multiple communication difficulties due to mental deterioration, multiple medication use, and reduced sensory abilities. Ethical assessment shows that dementia and Alzheimer’s disease patients lose their ability to make decisions, yet their medical treatment and sensory problems require caregivers to step in for consent and education, which does not follow established guidelines in everyday clinical settings [40]. The lack of proper communication between patients and staff members leads to medical errors because older patients with cognitive decline who do not maintain their oral hygiene will develop periodontal disease, which causes their cognitive abilities to deteriorate more rapidly through inflammatory mechanisms that affect both oral and neurological health [41].

Teach-back serves as an essential tool for this environment because it allows medical staff to confirm patient understanding while discovering hidden comprehension problems and enabling family members to support daily oral care practices. This method fails to receive consistent implementation because medical staff members face time restrictions and exhibit different levels of communication abilities. AI-augmented solutions solve these problems through automatic language reduction, multiple explanation formats, and their ability to monitor understanding over time, which follows cognitive changes that may occur in aging populations [95]. When properly framed, AI serves as a protective system that enhances teach-back education and family involvement in learning instead of taking over medical decision-making.

Tele-dentistry: restoring feedback loops in virtual care

Tele-dentistry provides patients with oral health care access but creates communication risks because patients cannot use body language, dentists cannot show physical examples, and patients cannot prove their comprehension in real time. Virtual dental services through tele-dentistry produce two main problems that users identify as poor understanding and weak communication between patients and dentists during their remote appointments for preventive education and self-care instructions [42]. The teach-back method works perfectly with tele-dentistry because it requires patients to repeat information through verbal communication. Research in oral-health education shows that teach-back methods enhance patient memory and treatment compliance because patients express their misunderstandings during active learning [15].

Medical staff working in remote areas do not have access to standardized assessment tools that enable them to evaluate patient comprehension. AI-based tele-dentistry systems provide these feedback systems through the ability to convert speech into text while evaluating semantic understanding and its automated clarification system, which activates during communication errors and produces documentation suitable for auditing purposes [43]. AI functions as a support system for dental professionals because it enables them to maintain their human connection with patients while providing accurate and dependable communication through virtual dental-care technology.

Public health dentistry: scaling comprehension beyond message exposure

Public health dentistry operates with an ongoing communication gap because people who encounter prevention messages fail to modify their behavior. Public health campaigns at the population level fail to address the different levels of literacy, cultural differences, and language obstacles, which lead to misunderstanding despite sufficient service availability. One study used mixed methods to identify major literacy deficiencies that affected patients who spoke other languages and those who did not complete their education when receiving dental care at multicultural public dental clinics [44].

Teach-back offers a mechanism to validate comprehension before scaling interventions. Research shows that teach-back programs administered by community health workers enable patients to remember health information better, while they build self-assurance and maintain their treatment plans during the first phase of treatment [45]. However, manual teach-back is difficult to standardize at a population scale. The public health dentistry field receives AI support to solve this problem through its ability to deliver information in multiple languages, automated semantic analysis, and the ability to enhance messages through actual comprehension data instead of tracking message distribution [96]. AI functions as a connection between mass communication and personal understanding, which allows dental health prevention programs to achieve equal results for all patients.

Equity, ethics, and health economics of AI-augmented teach-back

Health Literacy and Equity as Structural Determinants of Dental Outcomes

People experience ongoing dental health inequalities because they have different abilities to understand oral health information, and their language skills, educational backgrounds, and cultural environments vary. The process of teaching dental care for prevention and treatment requires specific guidance because standard counseling approaches assume patients will understand information in the same way. This assumption fails because analyses of clinics serving diverse populations show that patients do not understand these messages. Findings demonstrate that patients face challenges in understanding information because they receive messages but fail to grasp their meaning, and their literacy skills do not match the content, which creates a permanent barrier that exists independently of patient behavior [44]. The evidence focusing on practitioners confirms the existing gap because dentists report that their patients’ low health literacy, diverse cultural backgrounds, and ongoing misunderstandings create major obstacles for preventive care delivery despite their regular counseling efforts [97]. The existing differences among people lead to worse treatment adherence, and patients wait longer to get medical help, which results in different dental health outcomes for different groups of people.

Teach-back validation with AI functions as a structured system to solve present communication disparities that occur in dental offices by making staff verify patient understanding instead of presenting information. AI systems with educational capabilities provide users with explanations that match their reading abilities while supporting various languages and enabling patients to repeat information as many times as they want without facing time limitations or social judgment. The system achieves objective understanding verification through semantic analysis of patient restatements, which helps identify misunderstandings before they develop into clinical failures. The framework uses AI to enhance human empathy and clinical decision-making through its standardized system, which verifies patient understanding. The system links population-based messages to personal comprehension through its scalable solution, which minimizes dental care disparities caused by low literacy rates [96].

Multilingual AI and Culturally Accessible Communication

Dental care providers encounter ongoing equality difficulties because their current practice methods depend on family members who interpret languages and on unchanging translated written materials. These methods tend to neglect vital medical details about patient care requirements, risk severity, and treatment follow-through needs because they do not require confirmation of patient understanding. The existing restrictions lead to mistakes, together with cultural interpretations, which produce insufficient explanations during medical visits that may be complicated or stressful. Medical AI systems that support multiple languages have overcome their present limitations because these restrictions have stemmed from technological boundaries. The UNITED-MEDASR system uses synthetic data expansion together with semantic correction to provide accurate medical speech recognition across multiple languages, producing improved medical term recognition and lower transcription errors in actual medical conversations [86].

Conversational platforms that use multilingual avatars for speech recognition deliver active language changes, cultural message modifications, and direct user communication that exceeds the capabilities of fixed translation systems and enhances patient communication and understanding of medical information while enabling language-based comprehension checks [98]. The field of dentistry now uses AI communication tools that help dentists with diagnosis and treatment planning, while they improve patient understanding through natural language interfaces and decision support systems to reduce health literacy differences [99]. Evidence shows that multilingual AI systems perform translation and language-based comprehension verification that creates a necessary base for developing AI-powered teach-back models to serve all cultures and users equally.

Health Economics: From Communication Failure to Cost Avoidance

From a health-economic perspective, communication failure represents a substantial but often unrecognized cost driver in dentistry. Poor patient understanding leads to non-adherence, which results in missed appointments, preventable complications, retreatment, and avoidable emergency visits. An insufficient consent process leads to administrative problems and medical-legal fees. Downstream expenses mainly affect dental clinics that treat many patients and public dental care facilities because staff spend time teaching patients at the dental chair without achieving successful patient comprehension.

Digital health studies and tele-education research show that starting organized patient education programs at the beginning of care leads to financial benefits that often result in cost savings. A full analysis of digital innovation in oral health shows that mobile health tools, tele-dentistry platforms, and AI-based education systems help patients follow instructions better, which results in fewer dental procedures and decreased long-term expenses for dental care and office management [100]. A large systematic review and meta-analysis of telehealth interventions shows that telehealth programs generate positive cost-utility ratios because their emergency visit, hospital utilization, and overhead cost reductions surpassed their initial technology expenses [101]. Tele-education and remote communication tools enable health systems to achieve cost reductions in travel expenses, appointment wait times, and resource consumption, which result in financial savings throughout different health-care services [102-103].

Within this context, AI-augmented teach-back introduces only marginal per-patient cost while reducing failure rates, emergency utilization, and repeated chairside explanations. The AI teach-back system moves financial resources from fixing problems after they occur to enhancing patient understanding before treatment, which creates better economic results and produces superior health-care outcomes for all patients.

Ethical Risks, Governance, and Mandatory Safeguards

The ethical deployment of AI-augmented teach-back in dentistry requires explicit recognition of risks and the implementation of enforceable safeguards. Speech recognition systems create a major problem because they produce biased results that discriminate against people who speak with different accents and dialects. The World Health Organization (WHO) requires multimodal AI system outputs that include speech and language models to function as probabilistic information, requiring continuous bias evaluation and human supervision to prevent discriminatory communication breakdowns [87]. Automated systems encounter major issues because they generate simplified explanations that may use disrespectful language to address system users. The FUTURE-AI international consensus guidelines mandate designers to establish human interaction in AI systems to enable patients to control their information display settings for dignity, autonomy, trust maintenance, and AI system assistance without authoritative control [88].

Another essential risk emerges from data privacy issues and surveillance activities because AI-augmented teach-back systems operate through voice data processing, semantic analysis, and consent record management. Ethical frameworks have established three main requirements, including purpose limitation, patient consent for AI implementation, and dental practice control to stop data sharing and unauthorized patient profiling [89]. WHO guidance along with FUTURE-AI principles recommend that systems should perform privacy-protected local processing when possible, they need to track every action through detailed audit records, and they must provide understandable explanations about the assessment process to both medical professionals and their patients [87-88]. All these frameworks require AI-augmented teach-back to provide explanations and maintain audit trails without operating independently. The deployment of teach-back with bias mitigation, human oversight, patient control, and strong data governance systems enables better communication equity through AI augmentation to maintain dental practice ethical standards.

Implementation barriers and real-world challenges

Although AI-augmented teach-back is conceptually robust and well supported by communication science and educational theory, its translation into routine dental practice is inherently non-trivial. The dental field faces three main operational challenges: restricted appointment times, diverse digital capabilities in practices, and multiple legal requirements for patient consent, documentation, and information security. Implementation science suggests that communication innovations lose their effectiveness because they disturb current medical routines and create extra work for staff members. The process of scaling requires organizations to match their clinical objectives with the operational boundaries of dental practices, their financial rewards, and their organizational systems. *Time Burden and Workflow Disruption*

Clinicians find chairside teach-back to be an effective method, yet they believe it requires too much time and does not fit well into their busy schedules. Health-care facilities have shown that patient education tools and documentation systems create higher perceived workloads because they do not match existing workflows, resulting in extended patient visits and medical staff opposition [104]. The dental industry faces significant problems because different dental clinics operate at different digital levels and their technologies do not work together to optimize patient scheduling and treatment delivery [105].

However, ambient AI scribe implementations have started to demonstrate that properly built AI systems will reduce health-care staff administrative duties by automatically performing documentation tasks and cognitive work that medical staff would otherwise need to complete during appointments [106]. The teach-back method uses asynchronous AI delivery, which runs outside of patient appointments through a system that combines scheduling capabilities with dental software to enable clinicians to act as supervisors who verify information instead of teaching patients directly. The current system functions asynchronously, which means teach-back operates independently of workflows to provide support, which improves its operational viability while maintaining medical service standards [104,106].

Financial Barriers, Reimbursement, and Cost Uncertainty

AI-augmented teach-back provides excellent conceptual benefits, yet dental practices encounter authentic financial obstacles because most of their businesses operate as cost-conscious small to medium-sized enterprises. The system faces multiple issues, including the initial license expense, the need for equipment, maintenance costs, and an undefined payment method for AI-based patient education services. Economic data from related health-care fields show that these expenses will generate savings during future operations. Wu et al. conducted large-scale cost-utility analyses that demonstrate digital health interventions achieved favorable results by showing better early medical comprehension and reduced need for costly medical care in later stages of treatment [107]. Researchers who review telehealth and digital education systems have found that these technologies improve quality-adjusted life years, while they reduce both emergency department visits and hospital stays, proving their economic viability [108]. The implementation of clinical AI systems produces budget-impact results that show initial investment costs will be recovered through cost savings from improved processing efficiency, optimized workflows, and reduced medical errors [109]. The dental field can reduce barriers to adoption through cloud-based subscription models and per-patient pricing that allows AI-augmented teach-back to transfer costs from late-stage correction work to early-stage patient education.

Digital Literacy, Staff Adoption, and Trust in AI Systems

Successful implementation of AI-augmented teach-back faces challenges because dental teams display different levels of digital skills, and their trust in AI systems and workflow preparedness varies. Research into implementation shows that medical staff oppose digital solutions that modify their established procedures, demand long training periods, and produce unclear results that might result in limited acceptance, workarounds, and complete rejection [110]. A human-factors study shows that staff members may not participate in AI implementation because they lack AI knowledge, worry about ethical matters, and doubt AI effectiveness in clinical environments. However, training programs and governance systems can solve this problem [111].

Small and medium dental practices face challenges because they do not have sufficient technical backing to handle these operations. The deployment of AI systems requires three main mitigation methods: interfaces with basic training requirements, separate dashboard systems for dental staff and their assistants, and AI systems that produce clear explanations of results. Adopting AI tools requires short onboarding modules that deliver precise content and tools that assist users without fully taking over their work because these tools decrease mental work instead of making systems more complex [112].

Patient Trust, Acceptance, and Equity Considerations

Patient trust stands as a vital factor in determining successful implementation of systems in communities that show different levels of digital knowledge and health technology experience. Research shows that patients accept AI technology when it functions to support doctors through decision support systems that work to improve medical decision-making instead of taking away their clinical authority [113]. Users keep their trust conditional because they worry about privacy protection and algorithmic bias, and they fear AI systems will reduce their personal interactions with humans.

Survey results indicate that patients prefer human oversight of AI systems, and they want to challenge or stop AI-generated answers while receiving communication that respects their cultural background and personal requirements [114]. Research across multiple countries demonstrates that different regions show varying levels of acceptance, which is highest when there is open government oversight and effective regulatory systems. However, vulnerable populations continue to worry about data abuse and unfair system results [115]. The results demonstrate that AI-augmented teach-back requires a clear definition as a tool to support human health-care providers. However, it needs opt-out options, users must give direct consent, and the system should support multiple languages and various communication methods to build trust and achieve equal access for all users.

Data Governance, Privacy, and Regulatory Compliance

AI-augmented teach-back systems create major data governance obstacles because they require management of voice recordings, semantic comprehension logs, and digital consent records. Dental practices often operate as small independent units and may lack robust data-management systems, creating more chances for privacy violations and non-adherence to rules. WHO, in its 2024 guidance, requires health-care AI systems to maintain transparency while obtaining explicit consent, reducing data collection, and maintaining human control at all stages of data management [87]. Regulatory analyses that operate as complementary methods demonstrate that organizations need audit-ready data trails together with edge processing capabilities, local processing options, and practice-based governance systems to achieve trust and maintain accountability [116]. The design of AI-augmented teach-back needs to achieve assistive and explainable function and complete auditability because it should not operate independently as an autonomous system for safe dental practice integration.

Future research agenda and trial readiness

Researchers should conduct clinical trials on AI-augmented teach-back because its conceptual and technical aspects have reached their peak. The method has been proven feasible through technical assessments, and its ethical value has been validated by existing governance systems. It appears compatible with standard dental practice operations. The dental field lacks clinical evidence to prove the effectiveness of AI-augmented teach-back despite its strong conceptual support from communication science, digital health, and AI implementation research. Forward-looking research could measure actual oral health improvements and track patient progress over time. AI-augmented teach-back systems require validation through prospective research that employs standardized outcome measurement systems and implementation frameworks to assess their value, safety, and effectiveness in dental practices.

The first research priority needs to extend its work beyond the current focus on short-term memory. Most consent and patient-education studies assess comprehension at the point of care, yet dentistry requires sustained understanding of maintenance protocols, hygiene behaviors, and early warning signs of complications over extended periods. Teach-back methods enable patients to retain their knowledge while they follow medical instructions and prevent adverse incidents. However, the method fails in complete implementation because staff members experience difficulties with its application [13]. A surgical proof-of-concept investigation shows that teach-back methods help patients understand medical dangers and treatment procedures through improved collaboration with their medical team for decision-making [17]. The findings support the implementation of AI-augmented teach-back systems through a cluster-randomized controlled trial that should randomize dental clinics or providers to prevent treatment group contamination. The main outcomes need to measure patients' understanding after one month and six months using established semantic recall assessment tools and teach-back evaluation matrices that address the identified problem of sustained comprehension.

A second critical gap concerns the weak linkage between improved communication and demonstrable biological benefit. Dental communication research suggests a theoretical link between education and clinical outcomes, but direct dental evidence remains limited. The latest periodontal research shows that this connection produces significant measurable results. Analysis of long-term retrospective data demonstrates that patients who follow their supportive periodontal care regimen will experience minimal tooth loss while keeping their dental health stable, which proves that educational programs lead to better results because patients follow care instructions [36]. The results from prospective cohort studies show that the plaque index, gingival index, and bleeding on probing decrease at different rates depending on how severe the disease was at first and how well patients follow treatment instructions [117]. Research from observational studies shows that increased plaque levels and gingival inflammation lead to decreased oral health and quality of life, which supports the use of biological markers to predict patient results [118]. Future AI-augmented teach-back studies need to establish common biological assessment tools to track patient development across six to 12 months to establish clinically significant oral health progress [46].

Implementation science constitutes a third pillar of the future research agenda. Evidence from high-intensity clinical environments shows that algorithmic performance alone does not ensure adoption. AI tools used in critical care fail to exceed the 2% mark for clinical adoption because they do not function properly with medical workflows and they generate excessive work for health-care staff [119]. The dental industry operates under conditions that expose it to equal levels of risk as other industries. Organizations need to evaluate their delivery systems, including chairside-only operations, asynchronous (both pre- and post-visit) services, and hybrid service delivery models. Organizations should use asynchronous or hybrid work systems because these methods let them maintain medical checkups while enabling staff members to stay active. Workflow redesign literature states that clinical results need to be assessed together with consultation duration, documentation time, staff happiness, and patient discontinuation rates [120]. The research framework for AI-augmented teach-back trials requires teams to follow RE-AIM, CFIR, and NASSS frameworks while using SALIENT to direct AI system transparency management, accountability operations, and governance structures throughout system development [121]. Implementation outcomes should be treated as co-primary endpoints alongside patient comprehension.

Trial designs require direct equity inclusion because AI implementation does not automatically produce this advantage. Health-care AI research reveals that most AI systems fail to perform subgroup-specific outcome evaluations during clinical deployment [119]. Digital health equity research studies predefined equity targets and performs separate statistical evaluations instead of conducting unplanned statistical analyses [122]. Researchers need to assess digital health literacy because it functions as the fundamental element that determines how people engage with information and develop trust and understanding [123]. Researchers need to implement stratified randomization during future AI-augmented teach-back trials to separate participants based on their language abilities, educational backgrounds, age, and digital skills. The evaluation process needs to implement AI-aware implementation frameworks that include SALIENT, RE-AIM, CFIR, and NASSS to achieve scalability, maintain accountability, and perform clear equity assessments [121].

Finally, methodological innovation should be deliberately incorporated. Recent research shows that AI technology transforms clinical trial development by using adaptive systems that modify their methods based on real-time data analysis. AI-based systems enable real-time tracking through adaptive randomization and predictive modeling functions, which allow clinical trials to modify their operations while keeping their statistical integrity intact [124]. Conceptual frameworks show that systems that adapt need to operate under human monitoring, while organizations must follow regulatory standards to stop algorithms from creating both hidden and discriminatory systems [125]. Narrative synthesis shows that AI-specific methods, including reinforcement learning systems, decision-support systems, and digital twin implementation, lead to better operational efficiency and customized user experiences, but organizations need to create specific mechanisms that support system explainability and fairness for all users [111]. AI-augmented teach-back research needs to create specific rules to determine when the system should change, and all algorithmic operations must be documented, while doctors need to monitor the system to protect both scientific integrity and moral standards.

## Conclusions

This review reframes communication failure in dentistry as a modifiable clinical risk and positions AI-augmented teach-back as a credible pathway from passive patient education to verified clinical understanding. The article unites evidence from pediatric care with preventive dentistry, periodontal treatment, surgical methods, geriatric care, tele-dental services, and public health to show that manual teach-back enhances immediate understanding yet produces short-lived effects that fail to produce consistent changes in biological results or sustained benefits because of organizational obstacles including time constraints, differences among providers, insufficient tools to track understanding, and no effective systems for ongoing practice reinforcement. AI-augmented teach-back systems function as a standardized communication platform that enables audit capabilities and maintains equity standards through its combination of NLP with speech recognition, semantic understanding scoring, XAI, and risk-adaptive communication. The communication system allows medical staff to monitor patient care while solving operational challenges that exist in healthcare facilities. This review achieves its strongest point through its combination of communication science with implementation frameworks, including RE-AIM, CFIR, NASSS, and SALIENT; health equity research; and health economic data to demonstrate the clinical usefulness of AI-augmented teach-back in clinical settings and its readiness for trial implementation. The system encounters three major constraints: the absence of dental validation through direct prospective studies, inconsistent measurement of biological results, and the need for actual validation to show it works in clinical environments. This review maintains its focus on ethical safeguards, human-in-the-loop design, and governance requirements to prevent technological determinism from influencing the content. Collectively, this work advances the field by moving beyond whether teach-back “works” toward how verified understanding can be operationalized, measured, and sustained at scale. Future cluster-randomized, equity-aware trials linking comprehension verification to longitudinal biological outcomes are now essential to determine whether AI-augmented teach-back can deliver durable, equitable improvements in oral health care.
